# Neurofeedback training and motor learning: the enhanced sensorimotor rhythm protocol is better or the suppressed alpha or the suppressed mu?

**DOI:** 10.1186/s13102-023-00706-3

**Published:** 2023-07-31

**Authors:** Sana Afrash, Esmaeel Saemi, Anmin Gong, Mohammadreza Doustan

**Affiliations:** 1 Department of Motor Behavior and Sport Psychology, Faculty of Sport Sciences, Shahid Chamran University of Ahvaz, Ahvaz, 6135783151 Iran; 2 School of Information Engineering, Engineering University of People’s Armed Police, Xi’an, 710086 China

**Keywords:** Sensorimotor wave, Alpha wave, Mu wave, Motor learning, Novice golfer

## Abstract

A large number of previous studies have examined how different neurofeedback-based techniques may influence motor learning. However, only a few studies attempted to compare the effects of these different techniques on motor learning. Therefore, the present study attempts to examine the effects of neurofeedback training on motor learning in novice golfers, using three protocols, namely enhanced sensorimotor rhythm (SMR) at Cz, suppressed alpha waves at Fz, and suppressed mu waves at Cz. The participants were 64 adults (32 females; mean age = 22.31 ± 2.25 years). The study consisted of a pretest stage (day 1), intervention (6 sessions, over two weeks, 3 sessions per week), short-term retention (one day after intervention), and long-term retention (two weeks after intervention); in the pretest and short-term and long-term retention, motor performance for golf putting (12 trials) as well as amplitudes of SMR wave at Cz, alpha at Fz, and Mu at Cz were recorded. During each intervention session, the participants in three neurofeedback groups and a sham group first performed neurofeedback training (enhanced SMR at Cz, suppressed alpha at Fz, and suppressed Mu at Cz) for 20 min. Then, the participants in all groups performed three blocks of 12 trials consisting of golf putting training. The results indicated no difference between the sham and the experimental groups in the acquisition stage, as individuals in all groups experienced similar improvement in putting accuracy. However, in the short-term retention, all the three neurofeedback groups outperformed the sham group, although in the long-term retention, only the SMR group and the Alpha group showed a better performance than the sham group while the Mu group did not exhibit a notably better performance than the sham group. Our results also showed significant variations in the amplitudes of the SMR, alpha, and mu waves depending on the neurofeedback intervention provided, while no significant variation was observed in the sham group. Based on these results, it is recommended that coaches should make further use of enhanced SMR at Cz or suppressed alpha at Fz as their neurofeedback interventions to facilitate longer-term motor learning in golfers.

## Introduction

Since recent studies have demonstrated a connection between the brain and sports and cognitive functions [1], many researchers try to discover further details of this connection in an attempt to speed up the process involved in learning sports skills. In their studies, sports scientists have always sought methods that enable shorter learning times and greater sports achievements. Historically, athletes only focused on conventional training techniques designed to improve strength and endurance; however, current studies have clearly established influence of neurofeedback training on cognitive performance and psychological states in athletes [1]. By regulating brainwaves, neurofeedback training represents a fundamental training method that enables elite athletes and Olympians to achieve higher levels by improving their cognitive and motor performance [2]. In other words, brainwave control using neurofeedback training is an optimally quick way of reaching peak performance and enhanced learning in athletes [3]. Neurofeedback training can be described as a biological form of feedback [4]. Thus, many authors have focused on investigating brainwaves recorded through electroencephalography (EEG) for assessing performance in sports and tasks like golf putting, shooting, gymnastics, attention tasks, reaction time, and complex coordinated activities [5, 6].

Although clinical studies initially recommended neurofeedback training for treatment of psychological and cognitive disorders like attention deficit and hyperactivity disorder, depression disorder, anxiety, and post-traumatic stress disorder [7–11], later studies also pointed to potential applications of neurofeedback training for improving performance in healthy individuals, particularly athletes [3, 5, 12]. In other words, studies in this area have investigated neurofeedback training applications for improvements in such variables as motor learning and performance, increased concentration and attention, optimization of proper psychological states during execution and targeting, and they largely reported positive impacts of this method [1, 3, 13]. For example, Ros et al. [14] showed that during neurofeedback training sessions, dominance of a specific class of brainwaves may be associated with learning motor skills and improved sports performance. In fact, athletes during neurofeedback training teach their minds to control the activity of their brainwaves to achieve the best performance by suppression of waves above the standard range and enhancement of waves below the standard range [15, 16].

Brainwave frequencies cover the following bands: delta (0.5–4 Hz), theta (4–8 Hz), alpha (8–12 Hz), mu (8–13 Hz, at the central region), SMR (12–15 Hz), beta (13–30 Hz), and gamma (30–60 Hz). Suppression or enhancement of brainwaves at different ranges may lead to different results during neurofeedback training [17]. For example, SMR enhancement has been shown to considerably improve motor performance and learning in athletes [13, 18]. Furthermore, studies reported that alpha suppression training prior to motor performance intervention led to more positive results compared to alpha enhancement training [18]. Recent studies also demonstrated the role of mu suppression in improving efficiency of motor skills [5]. Moreover, Eschmann et al. reported that theta enhancement not only improved athletes’ motor performance, but also improved their cognitive control processes [19].

In the same vein, recent studies have compared different protocols proposed for neurofeedback training. For example, by comparing SMR enhancement and alpha suppression during shooting, Gong et al. showed that neurofeedback training based on SMR enhancement considerably improved shooting accuracy compared to the alpha suppression protocol [20]. In studying golf tasks, other authors found that neurofeedback training based on SMR enhancement during preparation for golf putting positively affected successful putting compared to a sham group [13].

Studies conducted using EEG have reported differences between skilled and novice golfers in terms of their brainwaves a few seconds before performing a putting task. For example, amplitudes of alpha waves (10–12 Hz) at Fz and Oz as well as mu waves (10–13 Hz) at Cz were smaller in skilled individuals than in novices while amplitude of SMR at Cz for skilled individuals were greater than the amplitude observed in novices [21]. In general, researchers argued that the type of protocol applied in neurofeedback training, the points where electrodes are placed, and the type of skill play an essential role in how this type of training may influence the results [20]. Studies have also showed that performance can be improved through each of SMR enhancement [13], alpha suppression [1], and mu suppression [5, 14, 21].

Although SMR and mu waves have almost the same range of frequencies, they are different in terms of their functions; mu waves (8 to 13 Hz; sensorimotor information) and SMR waves (12 to 15 Hz; somatosensory information) pertain to certain tasks associated with psychomotor processes involved in motor preparation [5]. However, researchers suggest that SMR enhancement is likely to indicate increased attention processing and, therefore, such enhancement during neurofeedback training is likely to lead to more successful golf putting [13]. The findings, however, are mixed. For example, some studies have suggested that while neurofeedback training with frontal alpha suppression in novice golfers can bring the patterns of their brain activity closer to those of skilled golfers, they still exhibit no considerable performance improvement over a control group [22]. Therefore, further studies are needed to better understand and identify the most effective neurofeedback intervention to improve motor learning. To the best of our knowledge, no study has been conducted so far to directly compare three neurofeedback interventions, namely SMR enhancement at Cz, alpha suppression at Fz, and mu suppression at Cz in a single study. Thus, the present study attempts to compare these three protocols in terms of their effectiveness in learning golf putting among novice golfers over six training sessions. Based on the existing literature [13, 20], we hypothesized that SMR protocol outperforms mu and alpha suppression protocols, leading to further motor learning.

## Materials and methods

### Participants

Gpower 3.1 was used to calculate the sample size for this study [23]. Based on the available literature [22], the data were analyzed using the following values; the sample size was calculated by assuming a significance level of 0.05, statistical power of 0.80, and average effect size of 0.33. A sample size of 20 was calculated using 4 (experimental groups) × 6 (sessions) mixed ANOVA. However, to improve statistical power and account for potential participant attrition, 64 adult individuals (32 females; mean age = 22.31 ± 2.25) were recruited for this study. We included individuals who (1) had no history of diseases; (2) were right-handed; (3) had no prior experience or proficiency in golf. In addition, we excluded individuals who (1) experienced any injury during the intervention sessions or (2) were not willing to continue participation in the study.

### Study design

As a study with quasi experimental design, the present study was confirmed by the university’s ethics committee and recorded (EE/1401.2.24.158645 /scu.ac.ir). All the processes involved in this study met the requirements of the Declaration of Helsinki, and the participants completed an informed consent form before taking part in this study.

### Apparatus

#### Golf putting

**Fig. 1:**
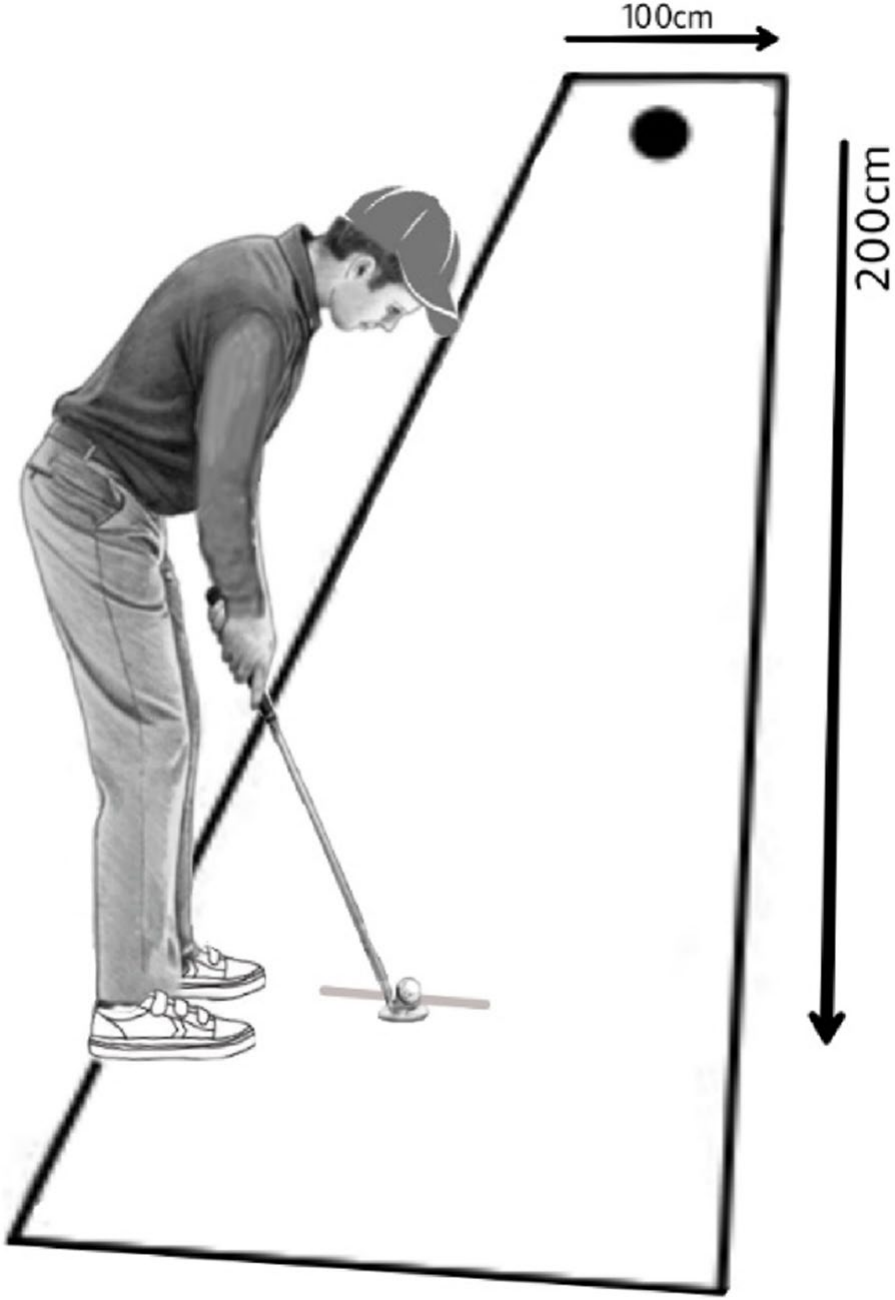
A schematic image of the golf putting task

We used a golf putting task proposed by previous studies [24]. The task involves a turf, a putter, and a golf ball. The participants used a standard right-handed putter for putting a standard golf ball measuring 4.27 cm in diameter across a turf measuring 400 cm by 100 cm. The turf included a target hole 10.8 cm in diameter placed 200 cm away from the start point. The start point was marked using a white strip 5 cm in width placed in the front of the putter. The distance between the ball and the center of the hole in each trial was recorded as a radial error to measure putting accuracy (Fig. 1).

#### Neurofeedback apparatus

**Fig. 2:**
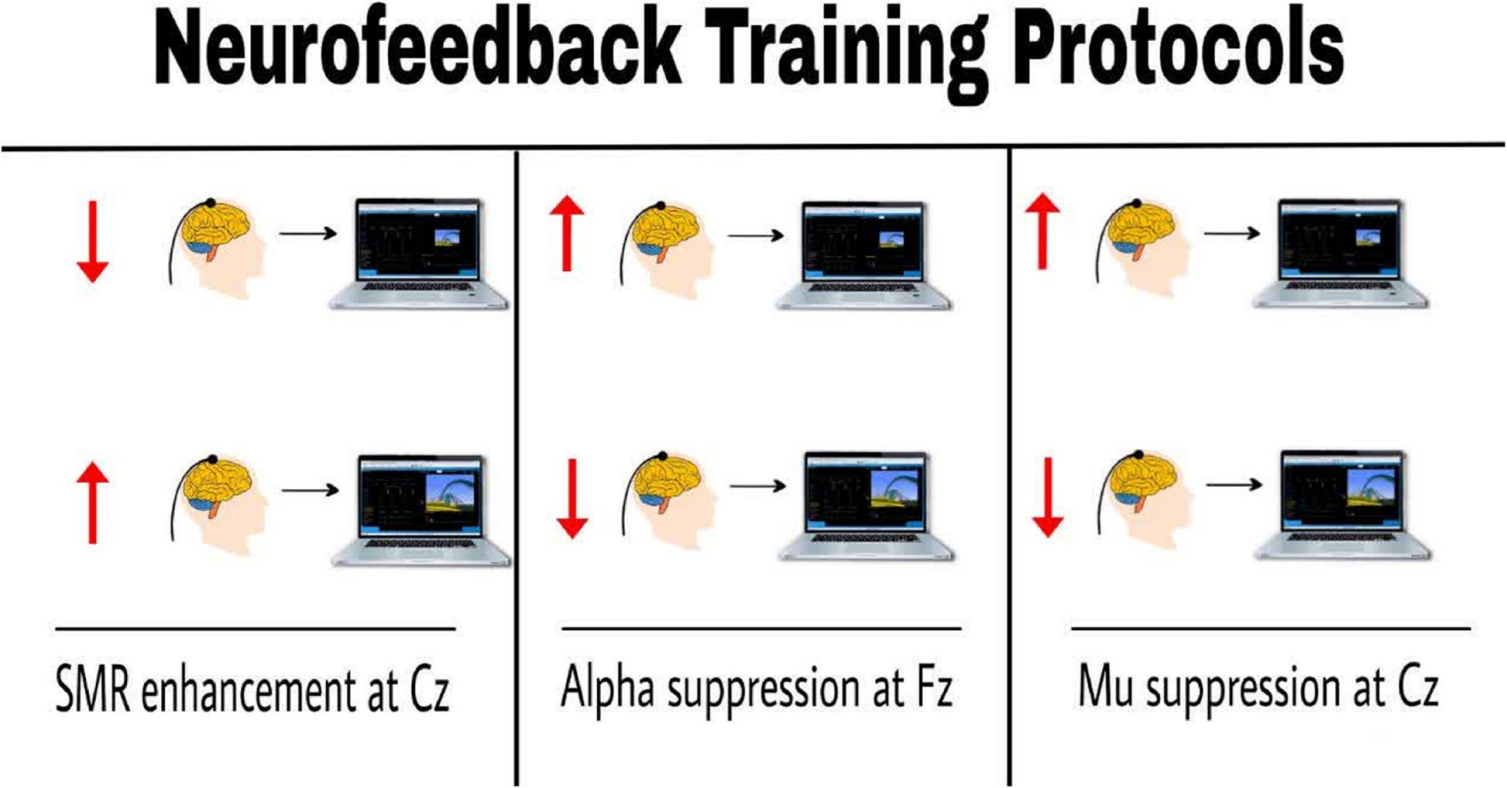
A schematic image for NFT program interface, in SMR enhancement protocol, with the decrease of the wave, the image on the monitor became smaller and with the increase of the wave, the image became bigger. However, for two other waves means Alpha and Mu suppression, with the increase of the wave, the image on the monitor became smaller and with the decrease of the wave, the image became bigger

Both neurofeedback training as well as brain waves records were conducted in a standardized fashion using the ProComp Infiniti 5 equipped with Biograph Software (ProComp Infiniti, Thought Technology; Montreal, Canada). In the beginning of the first session, brain waves (i.e. SMR and mu at Cz and alpha at Fz) were recorded for the participants under two conditions, i.e. with their eyes open and closed. Mean brain waves records were used to calculate the threshold for the training session. In order to remove some artifacts such as blinking, muscle contraction and other existing noises, in the device settings, a range of 5 to -5 for high artifacts and a range of 10 to -10 for low artifacts were considered, and noise and artifacts were removed visually. The ground electrode was connected to the left earlobe and the reference electrode was placed at the right earlobe. The brainwaves were received using gold-coated electrodes placed on the scalp. These were then converted to waves with different frequencies. All electrodes impedances were < 5 kΩ as well as the sample rate of the device for EEG data was 256 Hz. BioGraph showed these waves on a monitor (Fig. 2). The electrodes were placed on the scalp by an experimenter according to the 10/20 positioning system which is an internationally recognized method. During the training, the participant sat on a suitable wheelless chair in front of a computer monitor to watch a video. This enabled the subject’s constant concentration as well as provision of feedback from the system which together would initiate the neurofeedback training. According to Mirifar et al. [17] effective neurofeedback studies must contain a minimum of five training sessions at least one day apart. Other authors argued that three training sessions per week can lead to higher levels of effectiveness [25].

#### Procedure

**Fig. 3:**
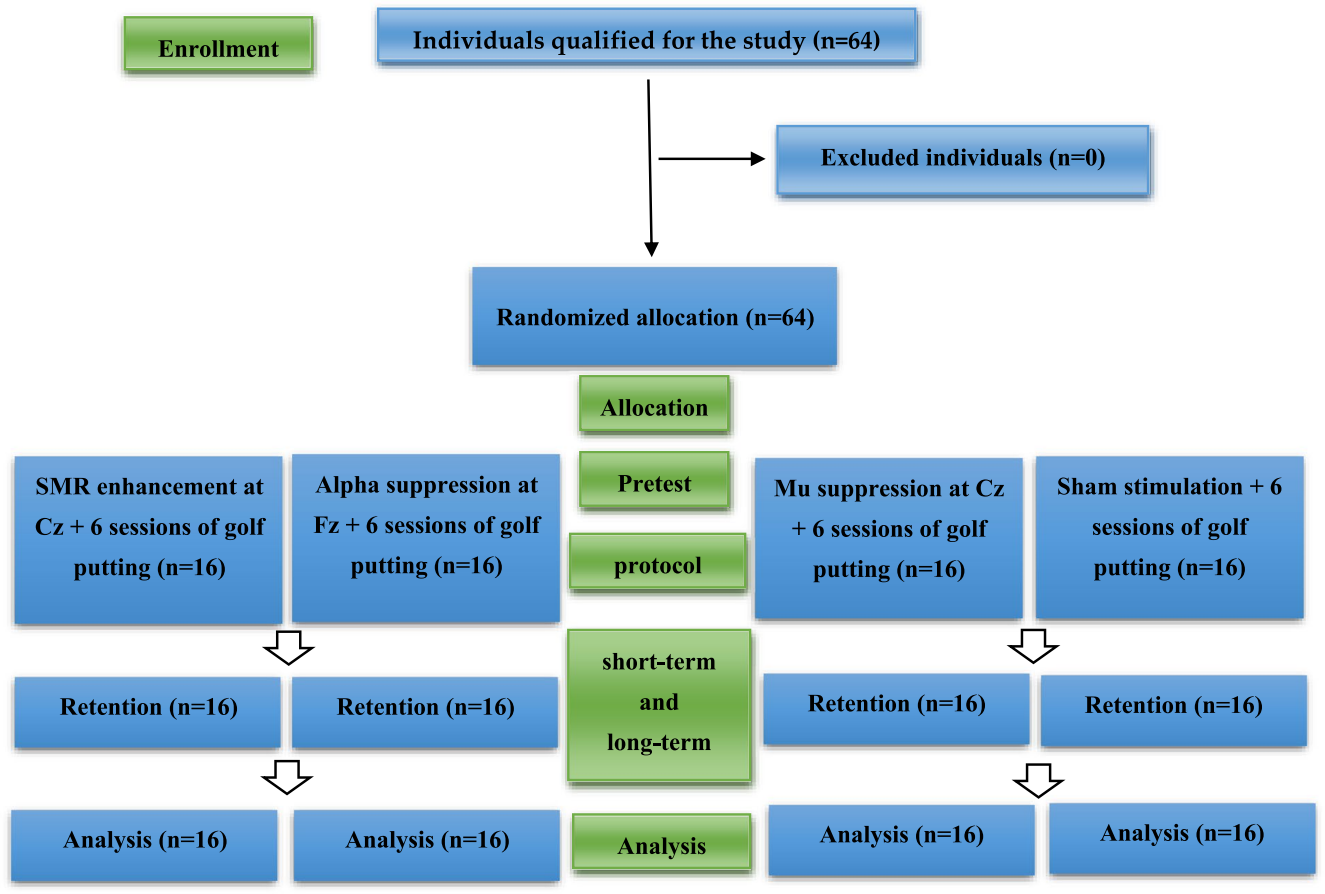
Flow diagram of the progress through the intervention

The present study was conducted using a quasi-experimental design. The participants were 64 adults (32 females) selected through convenient sampling and randomly assigned into four groups each consisting of 16 individuals (8 females in each group): (1) SMR enhancement at Cz (the SMR group), (2) alpha suppression at Fz (the Alpha group), (3) mu suppression at Cz (the Mu group), and (4) the sham group (individuals in this group performed the pseudo-training and received no actual neurofeedback). The participants were asked to stop drinking alcohol and using caffeine 24 h before the experiment day and recording of base waves. On day one, the participants were asked to give a short visit to the laboratory to see the equipment and apparatus. They were also given some explanations about the basic idea behind the study. The participants completed and signed an informed consent form. Moreover, to learn more about golf putting, the participants performed several golf putts on the turf as a form of mock preparatory stage. In the pretest, EEG data of participants were collected with eyes-closed for 2 min and eyes-open for 2 min. To control arousal levels, participants’ behavior and EEG signal quality were checked online in real time. Participants were asked to verbally cooperate if EEG revealed abnormal changes due to coughing, extra movements, etc. Next, the participants performed a test consisting of 12 trials of golf putting. The intervention consisted of six sessions organized over a period of two weeks, with three sessions per week. Each session involved 20 min of neurofeedback training in the form of watching a video. The intervention for the SMR group was designed in a way that would enlarge the picture viewed by the participant every time he or she could enhance the SMR (12–15 Hz) at Cz by improving accuracy and concentration while watching the video; through practice, it would become easier for the participants to enhance their SMR. Forthermore, a participant who could improve his or her performance in terms of accuracy and concentration, thereby causing lower brainwave errors, would also hear a feedback signal from the device which indicated good performance. As mentioned, the threshold were calculated based on the mean brain waves recorded on the first day and was set manually for each session and the participants could meet criteria approximately 60% the time [26]. The second group (the Alpha group) followed the same protocol, except that they would see an enlarged picture every time a smaller alpha wave was recorded. The picture was enlarged for the participants in the Mu group every time the recording device received a smaller mu wave (Fig. 2). As for the sham group, the participants watched a prerecorded video with variable picture size without any actual feedback for brainwaves. To ensure double blindness of the training, the experimenter only entered each participant’s unique ID number in the system [27]. The system would automatically identify the type of feedback for the participants and run the protocol accordingly. Therefore, the experimenter would have no knowledge of the type of feedback used in the training. Finally, following their respective neurofeedback training, all four groups practiced 36 golf putts consisting of 3 blocks of 12 trials in each session. One day following the completion of intervention, the participants performed one block of 12 golf putting trails together with a short-term retention test where their brainwaves were recorded with their bodies at rest and their eyes either closed or open and gazing. The long-term retention test was carried out using the same procedure but two weeks after the intervention (Fig. 3).

#### Statistical analysis

Central tendency and dispersion indices were calculated and diagrams were plotted using descriptive statistics. In addition, inferential statistical analyses were performed. For example; for motor acquisition index data, a 4 (experimental groups) × 6 (training sessions) mixed analysis of variance (ANOVA) was used. For motor learning index data as well as initial comparison of demographic characterizes between experimental groups a one-way ANOVA was used. Furthermore, for all pairwise comparisons, the Bonferroni post-hoc test was used. The data were analyzed at α ≤ 0.05 in SPSS 24 and the tables and plots were produced using Microsoft Excel 2016.

## Results

Initial analysis of the data confirmed normality and equality of variances. Table 1 reports the data on the study variables and characteristics of the participants. As seen in this table, in the pretest, all group had similar scores for the dependent variable “putting accuracy”.

**Table 1:** Characteristics of the participants

Characteristic	Groups (Mean ± SD)				Significance
	SMR	Alpha	Mu	Sham	
N	16	16	16	16	-
Age (year)	21.1 ± 87.78	22.2 ± 68.84	22.2 ± 81.37	21.1 ± 87.89	0.49
Height (cm)	170.5 ± 87.70	173.11 ± 50.39	172.9 ± 56.04	172.9 ± 56.89	0.88
Weight (Kg)	64.8 ± 56.35	72.12 ± 31.38	68.13 ± 62.22	71.13 ± 93.72	0.24
Putting accuracy (pretest, cm)	31.10 ± 42.49	29.6 ± 34.87	32.10 ± 00.58	30.8 ± 09.77	0.84

SMR: neurofeedback based on enhanced SMR at Cz; Alpha: neurofeedback based on suppressed alpha at Fz; Mu: neurofeedback based on suppressed mu at Cz; Sham: the sham group

### Motor acquisition

**Fig. 4:**
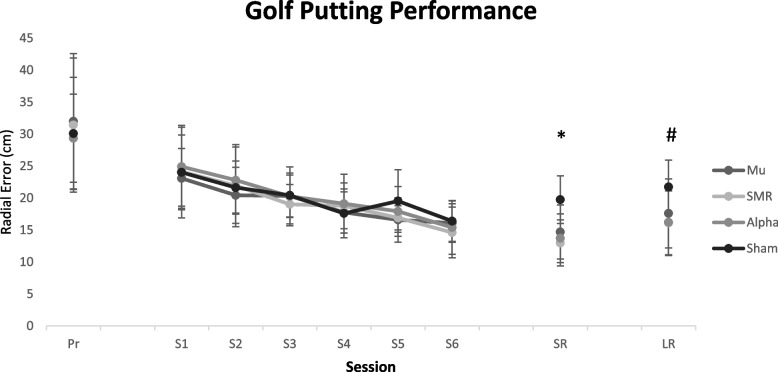
Line chart of golf putting accuracy during pretest, six sessions, short- term retention as well as long-term retention. Error bars represent standard deviations. Note. SMR: neurofeedback based on enhanced SMR at Cz; Alpha: neurofeedback based on suppressed alpha at Fz; Mu: neurofeedback based on suppressed mu at Cz; Sham: the sham group. Pr: pretest; S1 to S6: six training sections; SR: short-term retention; LR; long-term Retention. *Significant difference between all three neurofeedback groups with sham group; # Significant difference between both SMR and alpha neurofeedback groups with sham group, no significant differnec between mu neurofeedback group and sham group

Since significant values were found in Mauchly’s sphericity test (*p* < 0.05), Greenhouse–Geisser corrected values of intergroup Fs were reported instead. The results of the 4 (experimental groups) × 6 (training sessions) mixed ANOVA with repeated measures on sessions for radial error as the dependent variable during the acquisition stage showed that only the main effect for training session was significant (*F*(3.79, 227.42) = 55.62, *p* = 0.0001, partial η^2^ = 0.48). The results of the Bonferroni post-hoc test showed that the putting accuracy in the sixth session (15.61 ± .45) was significantly higher than the fifth (17.73 ± .46, *p* < .001), fourth (18.31 ± .48, *p* < .0001), third (20 ± .45, *p* < .0001), second (21.69 ± .63, *p* < .0001) and first (24.2 ± .75, *p* < .0001) sessions. In the fifth session, although the putting accuracy was similar to the fourth session (*p* = .99), it was significantly better than the third (*p* < .0001), second (*p* < .0001) and first (*p* < .0001) sessions. The scores of the fourth session were significantly higher than the scores of the third (*p* < .002), second (*p* < .0001), and first (*p* < .0001) sessions. Although there was no difference between the scores of the learners in the third session and second session (*p* = .053), these scores were better than the scores of the first session (*p* < .0001). The results also showed that the learners in the second session performed significantly better than the first session (*p* < .0001). In other words, all groups exhibited significant improvement in their performance during training sessions (Fig. 4). However, no significant values were found for other main effects of group (*F*(3, 60) = 0.37, *p* = 0.77, partial η^2^ = 0.01) or for group-session interaction (*F*(11.37, 227.42) = 1.02, *p* = 0.042, partial η^2^ = 0.04).

### Motor learning

In the short-term retention test (one day after the acquisition stage), the results of the one-way ANOVA indicated a significant difference between the experimental groups and the sham group in terms of radial error (*F*(3, 60) = 10.17, *p* = 0.0001, partial η^2^ = 0.33). In addition, the results from Bonferroni post-hoc test showed that all three neurofeedback groups, i.e. SMR (M = 12.97 ± 3.57), Alpha (M = 13.71 ± 3.76), and Mu (M = 14.69 ± 4.22), had radial errors that were smaller than that of the sham group (*p* = 0.0001, *p* = 0.0001, and *p* = 0.003, respectively). In other words, regardless of the protocol employed, all three neurofeedback groups experienced increased motor learning in the learners compared to the sham group (Fig. 4).

In the long-term retention test (two weeks after acquisition), the results of the one-way ANOVA showed a significant difference between the experimental groups and the sham group in terms of radial error (*F*(3, 60) = 4.45, *p* = 0.007, partial η^2^ = 0.18). The results of Bonferroni post-hoc test indicated that the SMR group (M = 16.12 ± 5.00) and the Alpha group (M = 16.21 ± 5.27) both had smaller radial errors compared to the sham group (M = 21.73 ± 4.20; both *p* = 0.01). No significant difference was found between the Mu group (M = 17.60 ± 5.40) and the sham group (*p* = 0.13). This means that only the learners in the SMR and the Alpha groups experienced increased motor learning (Fig. 4).

### Pre- and post-intervention changes in amplitudes

As seen in Table 2, brainwaves in all three experimental groups were significantly influenced by their respective protocols. In other words, SMR enhancement protocol was able to significantly enhance sensorimotor rhythm while the protocols used for alpha and mu suppression at Cz significantly suppressed the amplitudes of these waves. However, no significant difference between pre- and post-intervention amplitudes was reported in the sham group. This means that the neurofeedback interventions successfully achieved their intended goal; that is changing the brainwave amplitudes in the learners.

**Table 2:** Pre- and post-intervention amplitudes for the experimental and sham groups

Groups	Pre- and post-intervention amplitudes		Groups (M ± SD)		Significance level
			Before intervention	After intervention	
SMR	SMR wave amplitude at Cz	Open eyes	4.0 ± 91.94	5.0 ± 45.96	0.002*
		Closed eyes	5.1 ± 94.31	6.1 ± 50.27	0.004*
Mu	Mu wave amplitude at Cz (pretest)	Open eyes	10.2 ± 15.57	9.2 ± 11.74	0.0001*
		Closed eyes	19.4 ± 17.81	17.4 ± 22.38	0.003*
Alpha	Alpha wave amplitude at Fz (pretest)	Open eyes	8.2 ± 21.82	7.2 ± 13.35	0.001*
		Closed eyes	13.5 ± 81.90	12.5 ± 30.33	0.0001*
Sham	SMR wave amplitude at Cz	Open eyes	5.1 ± 13.11	5.0 ± 00.93	0.08
		Closed eyes	6.1 ± 05.40	5.1 ± 78.27	0.15
Sham	Mu wave amplitude at Cz (pretest)	Open eyes	8.2 ± 42.19	8.2 ± 62.38	0.34
		Closed eyes	14.4 ± 41.78	13.4 ± 98.67	0.11
Sham	Alpha wave amplitude at Fz (pretest)	Open eyes	7.1 ± 98.72	8.1 ± 13.96	0.48
		Closed eyes	12.3 ± 06.52	11.3 ± 53.21	0.07

SMR: neurofeedback based on enhanced SMR at Cz; Alpha: neurofeedback based on suppressed alpha at Fz; Mu: neurofeedback based on suppressed mu at Cz; Sham: the sham group

*Significant at *p* < 0.05

## Discussion

Our findings showed that although the neurofeedback training implemented using the three protocols, i.e. SMR enhancement at Cz, alpha suppression at Fz, and mu suppression at Cz, failed to indicate noticeable improvement in putting accuracy for novice golfers compared to the sham group in the acquisition stage, they successfully improved motor learning in the retention test. In other words, during the short-term retention test conducted one day after acquisition, participants under any of the three protocols outperformed the participants of the sham group and experienced improved motor learning. However, in the long-term retention test conducted two weeks after acquisition, only two neurofeedback groups (namely, the SMR and the Alpha groups) were able to retain this enhanced learning while the participants in the Mu group did not experience any positive outcome during this stage. These findings are consistent with a major part of the literature [13, 18, 20, 21, 24], highlighting the effectiveness of different neurofeedback protocols based on SMR enhancement or alpha or mu suppression in improving motor learning. For example, Pourbehbahani et al. [24] demonstrated that motor learning in novice golfers can be improved through six sessions of neurofeedback training based on SMR enhancement at Cz. Similarly, Wang et al. [21] reported positive effects of neurofeedback training involving mu suppression at Cz on learning golf putting.

In fine motor skills like golf putting, it is essential to maintain a good psychological state during a period that precedes execution of the skill in order to achieve a higher level of performance [28]. Since regulating the processes involved in motor programming while performing a skill can improve motor performance [29, 30], it is very important to identify new approaches to modification of motor programming processes to improve sports performance. Previous studies conducted using EEG have shown effectiveness of motor programming combined with brainwave control and regulation in motor preparation [30]. In addition, through a phenomenon known as neuroplasticity, neurofeedback training can establish new communication routes between brain cells at different regions of brain, and this plays an important role in enhancing memory, attention, and thereby motor learning [31].

Furthermore, the positive impact of neurofeedback training on motor performance can be explained based on the psychomotor efficiency hypothesis which proposes that such improvements involve a set of refined inputs to coordinate central neuromotor processes in the brain caused by suppression of irrelevant motor and cognitive preparation processes (for example, through attenuation of neuromotor noises) [32]. The hypothesis has been confirmed using the novice-expert model which shows that when performing different tasks like golf putts, shooting, archery shooting, and throwing darts, skilled performers outperform novices in terms of regulating their brainwaves according to models tailored to execution of such skills [13, 30, 33]. Thus, when an individual performs at a higher level, he or she experiences more stable psychological conditions. Regulation of brainwaves and bringing their amplitude to a desired range are among the factors that facilitate progress of athletes.

EEG-based studies have shown that brainwaves recorded from skilled individuals are different from those recorded from novices. For example, alpha waves at Fz and Oz as well as mu waves (8–13 Hz) at Cz in skilled individuals are smaller than in novices. In addition, SMR wave (15 − 12 Hz) at the sensorimotor cortex in the region Cz exhibited an increase a few seconds before a successful performance of putts [5]. Moreover, further studies by Wang et al. [1] examined brainwaves recorded during golf putts in skilled and novice golfers. They found smaller alpha (8–12 Hz) amplitudes at Fz-T7 and Fz-t8 as well as smaller mu amplitudes in skilled performers compared to novices.

In the same vein, researchers have found that during 8 sessions of neurofeedback training, individuals in an SMR enhancement group experienced positive impacts on golf putting in successful performance of putts compared to a control group. Furthermore, the authors attributed the increased SMR activity to improved attentional processing which led to more successful putts [13]. In addition, studies have demonstrated positive impacts of greater SMR amplitudes on improved performance of skilled shooters; researchers have also documented positive relations between improved visual motor performance and increased SMR activity [13]. SMR enhancement results in improved sensorimotor processing needed to maintain perception and attention; this can explain why enhanced SMR is related to lower levels of activity in thalamus nuclei [34]. Previous findings in connection to improved attention as a result of increased SMR activity Vernon et al. [35] suggest that these activities are closely related to optimization of motor performance as well as skilled performance in sports like golf and dart throwing. Moreover, greater SMR amplitudes are accompanied by lower activities in thalamus nuclei, which reduce interference with the sensorimotor processing needed for maintaining perception and attention [36].

Relatedly, Gong et al., [20] reported that six sessions of neurofeedback training based on SMR enhancement at Cz, C3, and C4 may lead to better performance compared to neurofeedback training based on alpha suppression at T3 and T4 in rifle shooters. This finding is inconsistent with our findings; we found similar results for SMR enhancement and alpha suppression protocols. The inconsistency between these findings can be attributed to the type of the task used. Studies have shown that effects of neurofeedback on complex tasks like those found in golf can be different from effects of neurofeedback on simpler tasks like shooting [21].

Our findings reported positive effects of different neurofeedback interventions on motor learning; however, these findings are not in line with a number of studies that failed to demonstrate such positive impacts. For example, Ring et al. [22] found that three sessions of neurofeedback training using a protocol involving suppression of alpha waves above the frontal strip and enhancement of SMR waves did not improve motor learning in novice golfers. Although they showed that neurofeedback training can to some extent reduce the differences between novice and skilled individuals in terms of the pattern of alpha waves in their brains, the process used in their study failed to improve motor performance. Specifically, Ring et al. [22] found that in a mental task involving assessment of training difficulty, the participants in the alpha group assessed the training to be significantly more challenging compared to the participant in the SMR group. This shows that SMR feedback can be perceived as more convenient form of feedback by participants.

In addition, the participants in the present study performed the neurofeedback training over two weeks (three sessions per week). Previous studies have shown that three sessions of training per week can bring about more positive effects than protocols that involve two sessions of neurofeedback training per week [25]. Therefore, the positive effects of the neurofeedback interventions used in the present study can be attributed to the fact that we had (not less than) three sessions per week. Moreover, we included a sham group to perform neurofeedback training without actual feedback while the participants did not know which intervention they have been assigned to; this practice eliminated potential biases and suggestions. Thus, our study represents a better practice than the one used in previous studies that lacked a sham group [37]. Like any other study, our study had a number of limitations, too. For example, we did not include a control (no intervention) group and, therefore, we might not have been able to fully account for effects of neurofeedback training on acquisition of golf putting skill. We recommend that future studies should add an actual control group to their design in order to clearly account for such potential effects. Furthermore, we only investigated the individual impact of each neurofeedback intervention on motor learning, without examining their combined effects. It is possible that a combination of two or three protocols can produce more stable positive effects. Thus, it is recommended that future studies include mixed groups to further examine the potential combined effects of these protocols.

## Conclusions

In summary, our results indicated that all three neurofeedback interventions, namely SMR enhancement at Cz, alpha suppression at Fz, and mu suppression at Cz, can improve short-term motor learning. However, when it comes to longer term motor learning – particularly for golfers – the individuals who received SMR enhancement or alpha suppression outperformed the participants who were subjected to mu suppression. Therefore, it may be recommended that golf coaches should try to make further use of neurofeedback techniques, particularly SMR enhancement or alpha suppression protocols, when teaching golf.

## Data Availability

The datasets used and/or analysed during the current study available from the corresponding author on reasonable request.
